# Electrophysiological Assessment of Semantic Processing of Cochlear Implant Users Using an Audiobook

**DOI:** 10.1177/23312165261439202

**Published:** 2026-04-27

**Authors:** Shimin Mo, Claude Alain, Kristen E. Li, Andrew Dimitrijevic

**Affiliations:** 1 Institute of Medical Science, Temerty Faculty of Medicine, 12366 University of Toronto, Toronto, ON, Canada; 2 Evaluative Clinical Sciences Platform, 282299 Sunnybrook Research Institute, Toronto, ON, Canada; 3 Rotman Research Institute, Baycrest, Toronto, ON, Canada; 4 Department of Psychology, 215445 University of Toronto, Toronto, ON, Canada; 5 Music and Health Research Collaboratory, Faculty of Music, 177422 University of Toronto, Toronto, ON, Canada; 6 Department of Physiology, 233839 University of Toronto, Toronto, ON, Canada; 7 Neurosciences and Mental Health, 7979 Hospital for Sick Children, Toronto, ON, Canada; 8 Department of Otolaryngology-Head and Neck Surgery, Temerty Faculty of Medicine, 177422 University of Toronto, Toronto, ON, Canada

**Keywords:** semantic processing, ecological validity, cochlear implant, temporal response function, EEG (electroencephalography), N400, speech tracking

## Abstract

Cochlear implants (CI) afford listeners with limited spectral resolution, thereby constraining semantic processing. Prior research has predominantly used isolated words or sentences without context, a method that lacks ecological validity and may place greater demands on working memory (WM) due to the absence of contextual linguistic information. This study examined the semantic processing of experienced CI users (*N* = 21) and age-matched normal-hearing controls (NH, *N* = 21) using an audiobook. We measured neuroelectric brain activity while participants listened to a story in which we inserted an incongruent word every 45 s, herein, the E45 condition. WM and behavioral semantic judgment were quantified with a listening span test. Temporal response functions, linear models that characterize how continuous stimulus features are temporally mapped onto neural responses, were used to quantify speech neural tracking of audio features (envelope, word onset, word surprisal) during ongoing speech. Results showed reliable neural tracking of acoustic features and word surprisal. Compared to the Control condition, E45 elicited larger TRF-N400 responses in both groups. Although TRF-N400 amplitudes were comparable between CI and NH listeners, CI users exhibited delayed responses. In NH listeners, larger N400 effects were associated with greater semantic judgment accuracy, linking neural responses to behavioral performance. These results show the validity of an ecologically valid paradigm that does not require an explicit behavioral response to quantify semantic processing in CI users.

## Introduction

A cochlear implant (CI) is a device that provides auditory input to individuals with severe to profound hearing loss. It has long been recognized that CI outcomes vary significantly; that is, not all CI users benefit equally from their device (McRackan et al., 2018). This variability is reflected in measures of CI outcomes, such as objective speech perception tests and subjective perception of quality of life questionnaires (e.g., Patient-Reported Outcome Measures, PROMs). A longitudinal study tracking speech perception changes after CI reported substantial variability across 100 postlingually deafened adult CI (Lazard et al., 2010). Specifically, at one year post-implantation, word and sentence recognition scores ranged from 0% to 100%, highlighting the pronounced variability in speech perception outcomes within this population. Likewise, 13% of CI users were still considered poor performers one year post-implantation in 445 adult CI users (Bodmer et al., 2007). These poor performers either did not improve, performed worse, or showed an improvement of less than 10% in speech perception tests. Given this substantial heterogeneity in outcomes, a critical question is what mechanisms drive such variability across CI users. The following section outlines two major factors contributing to the variability in CI outcomes (Moberly et al., 2016).

A CI has both an external and an internal component for transmitting auditory information. The external component consists of a microphone that receives auditory input, which is then converted into electrical signals by a speech processor. The electrically encoded signal is transmitted to the internal receiver, which passes it to electrode arrays within the scala tympani of the cochlea and sends the electrical signals to the cochlear nerve fibers. Although CI manufacturers have significantly improved their technologies, restoring natural hearing through CI is far from perfect (Naples & Ruckenstein, 2020). Specifically, the CI provides limited spectral resolution due to the insertion length of the electrode array, the number of stimulating electrodes, and the spread and overlap of excitatory current from the CI electrodes. First, the electrode array inserted into the cochlea has a limited insertion depth and is not inserted far enough to cover the entire apex, as deeper insertion is associated with higher trauma incidence rates (Hrnčiřík et al., 2024; O’Connell et al., 2016). As a result, the frequency range of the incoming auditory signals must be assigned to electrodes that do not reach the cochlear apex, causing a spectral misalignment between the acoustic input and the electrode positions within the cochlea (Svirsky et al., 2015). Second, although the electrode array typically has around 20 stimulating electrodes, the number of independent information channels transmitted by the implant is limited to roughly four to seven (Friesen et al., 2001). This is due to the spread of excitation between adjacent electrodes, causing overlapping regions of neural stimulation. As a result, individuals with CIs receive speech input that is both spectrally shifted and degraded (Fu et al., 2005), which contributes to variability in speech recognition and leads to poor outcomes (Moberly et al., 2016).

However, degraded sensory input alone does not fully account for why some individuals achieve better outcomes than others. Given the degraded quality of auditory input from a CI, relying solely on bottom-up information can lead to poor speech perception. Top-down processes can help compensate for these limitations and support speech understanding. Factors such as linguistic knowledge (phonological, lexical, and semantic knowledge) and cognitive abilities are commonly studied in this context, as they play a crucial role in facilitating comprehension (Moberly et al., 2016). To access linguistic knowledge and retrieve a reasonable lexicon, a prerequisite is being able to hold the heard information in mind. Thus, cognitive factors such as working memory (WM) play a crucial role in speech understanding. In older adults with varying degrees of hearing loss, the semantic context benefits of spoken-word recognition are modulated by verbal WM and age, and WM is one of the consistent predictors of listening performance (Janse & Jesse, 2014). For people with CIs, a greater WM span is associated with a higher subjective quality of life (Prince et al., 2024) and better speech recognition (Hua et al., 2017; Moberly et al., 2017). The Ease of Language Understanding (ELU) model (Rönnberg et al., 2013) posits that normal hearing (NH) adults can automatically and rapidly match incoming speech (if not degraded due to environmental factors) with the stored phonological representations. However, for people with hearing loss or CI users, the degraded speech signal leads to a mismatch with the phonological representations, requiring more top-down cognitive processes to resolve the mismatch. That is, hearing-impaired listeners need to hold the information in WM while a processing loop uses phonological and semantic long-term memory information to fill in the missing information. Hence, people with better WM are better able to retain more information in mind before the processing loop settles on an appropriate answer if auditory input is degraded.

Beyond behavioral measures of speech recognition, recent advances in neurophysiological methods have made it possible to directly examine how the brain tracks and processes continuous speech. Recent advances in signal processing and analysis methods have enabled researchers to model neural responses to complex, continuous stimuli. This methodological development makes it feasible to use more ecologically valid materials, such as continuous speech (e.g., audiobooks), which better approximate real-life listening experiences and provide insight into speech processing in participants’ daily lives. The temporal response function (TRF) is a modeling method that quantifies the mapping between continuous speech and neural responses to the speech. It has been widely used as a measure of neural speech tracking, reflecting a mapping between audio features and neural responses (Ding & Simon, 2014; Obleser & Kayser, 2019; Xiu et al., 2022; Panela et al., 2024).

TRF can quantify neural tracking of low-order acoustic features, such as the broadband temporal envelope of speech, and higher-order linguistic features, like word surprisal (Gillis et al., 2021; Weissbart et al., 2020), which reflects the conditional probability of each word given its preceding lexical context in the continuous speech stream. For instance, Gillis et al. (2021) derived word surprisal values from a computational language model applied to continuous stories and included them as predictors in a TRF framework while controlling for acoustic features. They found that surprisal explained unique variance in neural responses beyond that accounted for by acoustic tracking in young NH adults. Thus, by modeling neural responses as a function of changes in linguistic features, such approaches provide a way to quantify higher-order speech processing.

The TRF has been successfully recorded in a limited number of CI studies (Nogueira & Dolhopiatenko, 2022; Paul et al., 2020; Verschueren et al., 2019; Xiu et al., 2022), demonstrating that speech neural tracking can be reliably measured in CI users.

One well-established neural marker of higher-order semantic processing is the N400 electrophysiological component, which provides a means to study semantic processing (Kutas & Hillyard, 1980). It is a late negative wave that occurs between 200 and 800 ms after stimulus onset, typically observed in the central-parietal scalp region. The N400 amplitude is linked to the degree of semantic incongruency in the stimuli, with greater semantic incongruency resulting in increased negativity.

In both NH and CI listeners, the N400 effect tends to be smaller in amplitude or delayed in onset when the intelligibility of the input is reduced. For instance, Obleser and Kotz (2011) manipulated sentence intelligibility using 1-, 4-, and 16-band vocoded speech and found that the N400 effect became stronger and occurred earlier as intelligibility improved. While there are comparatively fewer studies examining the N400 in CI users, the available evidence points to a similar pattern: CI users show sensitivity to incongruency during semantic processing, but with longer latencies relative to NH listeners (Deshpande et al., 2024; Finke et al., 2016; Hahne et al., 2012). Taken together, these findings suggest that N400 amplitude is largely comparable between CI and NH groups, but its latency is significantly delayed in CI users.

Recent evidence from naturalistic speech-tracking suggests that these changes in the N400 may reflect a shift in how the brain utilizes different levels of linguistic prediction. Broderick et al. (2021) dissociated the N400 response into two distinct contributions: lexical surprisal (the probability of a specific word identity) and semantic dissimilarity (the degree of meaning change). Their findings showed that in older adults, lexical surprisal responses remain robust, albeit delayed, whereas responses to semantic dissimilarity are markedly reduced. This dissociation suggests a shift in predictive strategy: the aging brain may maintain relatively rigid predictions about specific lexical items while showing diminished flexibility in preactivating broader semantic categories (Broderick et al., 2021).

Such a dissociation provides a potential framework for understanding the delayed N400 latencies observed in CI users. The reliance on preserved, rigid lexical predictions may serve as a compensatory strategy to navigate corrupted auditory input with limited cognitive resources. However, if these rigid predictions are contradicted by the incoming signal, the resulting neural reevaluation may manifest as the characteristically delayed N400 latency. Thus, the N400 in CI users may not only reflect a sensory delay but also a fundamental reorganization of top-down predictive hierarchies in response to chronic auditory degradation.

Importantly, although prior studies employed different experimental paradigms, none reflect the type of listening experiences CI users encounter in daily life. Deshpande et al. (2024) employed a triplet-digit task in which participants judged whether digit sequences followed a congruent or incongruent ascending/descending order; Finke et al. (2016) used a word-odd task requiring detection of living entities among nonliving ones; and Hahne et al. (2012) implemented a sentence congruency judgment task to assess sensitivity to semantic violations in sentences. Unlike these paradigms, CI users often have to attend to long and complex sentences in a daily listening environment. Thus, the ability to grasp the context and gist of the speech is crucial for their comprehension. Traditional paradigms using single words or sentences do not offer as rich contextual information as running speech, such as an audiobook, making it difficult to assess how CI users process semantic information in a real-life context.

Additionally, many prior studies on CI users have focused on identifying single words. In this study, we assess semantic processing in CI users using an audiobook and examine how semantic processing in more naturalistic situations comprises linguistic contextual cues related to WM. This more ecological approach offers an objective neural alternative to traditional speech intelligibility testing without requiring explicit behavioral responses. While successful neural tracking still depends on sustained attention to the stimulus, the paradigm does not rely on overt task performance. Beyond revealing individual differences in speech comprehension, it may provide valuable insights for post-CI rehabilitation and inform recommendations for auditory training. This approach can also help transform audiological and clinical practice by enabling reliable assessments in populations for whom behavioral responses are difficult to obtain, such as young children, individuals with cognitive impairments, or patients unable to provide consistent feedback.

In this study, we quantified the semantic processing of naturalistic speech using the N400 component. Although prior studies generally report comparable N400 amplitudes but delayed latencies in CI users, these findings are based on highly controlled, short-duration tasks with limited contextual demands. Processing continuous speech through degraded CI input is challenging and places greater demands on contextual integration and sustained processing (Peelle, 2018), potentially altering both the timing and magnitude of semantic responses. Therefore, we hypothesized that CI users would exhibit delayed N400 responses and potentially reduced amplitudes relative to normal-hearing (NH) controls. Moreover, we hypothesized that WM is positively correlated with the TRF-N400 response, such that people with greater WM will show a stronger and earlier TRF-N400 response than those with lower WM.

## Method

This study adhered to the STROBE guidelines for reporting cross-sectional studies (von Elm et al., 2008).

### Participants

Demographic information for all CI participants is presented in Table 1. Twenty-one CI users were recruited from the Department of Otolaryngology at Sunnybrook Health Sciences Center. The age of CI participants ranged from 57 to 80 years (*M* = 70.3, *SD* = 7.3), comprising 12 females and 9 males, all of whom were without neurological conditions. The CI group included 5 bilateral users (implanted in both ears), 5 unilateral users (implanted in either the left or right ear), and 11 bimodal users (had a unilateral CI combined with a hearing aid in the contralateral ear). Hearing-related demographic measures included age at implantation, age of deafness onset, and total duration of deafness, which was calculated by subtracting the reported onset of deafness from the date of implantation. These measures, together with outcomes from online assessments and surveys, were used in correlational analyses. All CI participants were adults with postlingual hearing loss. A total of 21 age-matched controls were recruited, aged 63 to 81 years (*M* = 72, *SD* = 4.9), including 11 females and 10 males. All were native English speakers and reported no history of neurological conditions such as stroke or dementia. All participants underwent pure-tone audiometry to verify normal hearing, defined as thresholds below 25 dB across octave frequencies from 250 to 8000 Hz. Written informed consent was obtained from each participant. An independent samples Welch's *t*-test indicated that age did not significantly differ between the CI group (*M* = 70.33 years) and the NH group (*M* = 72.05 years), *t*(35.19) = −0.89, *p* = .378, A chi-square test of independence showed that sex distribution did not differ between groups, χ^2^(1) = 0.00, *p* = 1.00. The study size was determined by participant availability during the recruitment period rather than by an a priori sample size calculation. A sensitivity analysis conducted in G*Power (Faul et al., 2007) for a one-tailed independent-samples *t*-test (α = .05, power = .80; *n* = 21 per group) indicated that the minimum detectable effect size was Cohen's *d* = 0.78. That is, this study was sufficiently powered to detect large between-group effects (*d* ≈ 0.8) but may have been underpowered to detect small-to-moderate effects (0.2 < *d* < 0.5).

**Table 1.** CI Participants Demographics.

ID	Age (Years)	Sex	Duration of deafness	Year of CI Use	Implant Side	Etiology	CI Brand	AzBio	Number of Electrodes Blocked
CI01	77	M	31	12	Bilateral	Unknown	MED-EL	93	4
CI02	79	M	15	14	L	Meniere's disease	MED-EL	87	3
CI03	77	M	24	8	Bilateral	Unknown	MED-EL	79	4
CI04	73	M	49	6	Bilateral	Unknown	MED-EL	94	4
CI05^a^	78	M	10	7	L	Unknown	MED-EL	93	3
CI06	57	F	6	14	L	Unknown	MED-EL	94	3
CI07	72	F	40	12	L	Unknown	AB	93	4
CI08	65	M	20	7	Bilateral	Hereditary	MED-EL	86	4
CI09^a^	63	F	11	8	R	Unknown	MED-EL	53	4
CI10	66	F	7	22	Bilateral	Hereditary	Cochlear	93	3
CI11	73	M	48	5	R	Unknown	Cochlear	97	2
CI12^a^	67	F	24	3	R	Unknown	AB	94	4
CI13^a^	65	F	14	6	L	Meniere's disease	Cochlear	97	4
CI14^a^	59	F	39	4	R	Unknown	MED-EL	93	2
CI15^a^	74	F	21	2	L	Unknown	MED-EL	90	3
CI16^a^	64	F	2	4	L	Unknown	AB	100	4
CI17^a^	80	M	28	2	R	Meniere's disease	AB	64	4
CI18^a^	68	M	51	15	R	Unknown	AB	85	3
CI19^a^	79	F	23	16	R	Hereditary	AB	82	4
CI20	62	F	25	3	R	Hereditary	MED-EL	90	3
CI21^a^	79	F	15	2	L	Unknown	Cochlear	96	3
**Average (*SD^b^*)/Count**	**70.3 (7.3)**	**F:12, M: 9**	**24 (14.5)**	**8.2 (5.6)**	**L:8, R:8, Bilateral: 5**			**88.2 (11.3)**	**3.4 (0.6)**

a Bimodal (CI + HA) users.

*Note*: The bold text in last row summarizes the average (standard deviation) and count of the demographic characteristics. *SD*: standard deviation.

### Listening Span Test

Working memory was assessed using a listening span task adapted from Prince et al. (2024). The auditory stimuli were played aloud through a speaker. On each trial, participants heard a series of sentence–word pairs. For each sentence, they judged whether it was semantically plausible by selecting “Yes” or “No” on a computer. For example, a plausible sentence might be, “He ran out of money, so he had to stop playing poker,” while an implausible one could be, “The acid is so big that it doesn’t fit in the parking lot.” After selecting “Yes” or “No” for sentence plausibility, participants heard a single word (e.g., “torch”) presented audibly for 950 ms. They were asked to memorize these words and recall them at the end of the trial. The number of sentence–word pairs presented before recall (i.e., memory load) ranged from two to six pairs. After the final pair in each trial, participants were prompted to type all the words they could remember using the computer keyboard. Each load size was presented three times, resulting in a total of 15 blocks. Participants also completed two practice blocks, during which they were asked to adjust the speaker volume to a comfortable level. Performance was measured by the total number of words correctly recalled and the total number of correct sentence judgment across all blocks.

### Speech, Spatial, and Quality of Hearing Survey

The speech, spatial, and quality of hearing (SSQ) questionnaire is a validated (Gatehouse & Noble, 2004) and reliable self-reported measure for CI users (Tyler et al., 2009). It evaluates three key domains of hearing: speech hearing (understanding speech across different listening situations), spatial hearing (perceiving the direction, distance, and movement of sounds), and qualities of hearing (sound segregation, recognition, speech clarity, and listening effort). Each SSQ domain contains a different number of items: 14 for speech hearing, 17 for spatial hearing, and 18 for qualities of hearing. Responses are rated on a scale from 0 to 10, with higher scores indicating better perceived hearing ability. For each domain, scores were averaged to obtain a mean rating. Both CI users and TH controls completed the SSQ.

### Electrophysiological Recording

Electroencephalogram (EEG) data were collected using a 64-channel antiCHamp Brain Products system (Brain Products GmbH, Munich, Germany) with a sampling rate of 2,000 Hz. Participants wore EEG caps with electrodes positioned so that the one corresponding to the standard Cz location, according to the 10–20 international system, was placed at the vertex of the skull, determined by the intersection of nasion-to-inion and tragus-to-tragus midpoints. A separate reference electrode, positioned slightly anterior to the vertex, served as the reference, while the ground electrode was placed on the midline, halfway between the nasion and vertex. Electrodes near or over the CI magnet and coil were excluded. Around 2–4 electrodes were blocked off per CI. Bimodal CI users were tested in their everyday listening configuration and were allowed to use their contralateral hearing aid during the experiment.

#### Audiobook Listening Task

Each participant listened to an audiobook (“Diary of a Wimpy Kid”) for four runs, each lasting approximately 15 min. Two runs were control runs. For the other two runs, a word from the original audiobook script was replaced by a surprising word roughly every 45 s, referred to as the E45 condition. For example, in the sentence “John actually got ahold of my last journal a few weeks back and it was a disaster, but don't even get me started on that story,” the last word was replaced with “chinchilla”. The order of conditions was counterbalanced across participants. Importantly, participants never listened to the same story content under different conditions.

Surprisal values were generated from a 5-gram model trained on the Corpus of Contemporary American English (Davies, 2010) using the KenLM tool (Heafield, 2011). In this framework, surprisal is defined as the negative log probability of each word given the four immediately preceding words, providing a direct and interpretable information-theoretic estimate of lexical predictability. Restricting context to a fixed n-gram window avoids introducing additional assumptions about how contextual influence should be weighted (e.g., decay functions or tunable window parameters), thereby maintaining transparency and reproducibility of the probabilistic estimates. All words in the audiobook had a surprisal value that reflects how unexpected the word is in the context of the preceding four words. The surprisal *S* of experiencing word *w* at time point *k* (*w_k_*) is inversely related to the probability (*p*) of *w_k_* given the four preceding words (Levy, 2008). A more surprising word has a higher surprisal value. These surprisal values were included as a predictor in the TRF model alongside acoustic features. Model comparisons were then used to assess whether adding surprisal improved the prediction of neural responses beyond that explained by acoustic features alone, thereby quantifying the unique contribution of higher-order linguistic processing. More recent studies have employed transformer-based language models (e.g., GPT-2), which leverage long-range contextual information through attention mechanisms (Boeve & Bogaerts, 2025; Momenian et al., 2024; Xie et al., 2025). The present study, however, was designed and implemented at a time when n-gram models remained the predominant approach for estimating lexical surprisal in neural research (Brodbeck et al., 2022; Xie et al., 2023).

Criteria for word substitution were: (a) noun for noun; (b) at the end of a sentence or a clause; (c) no grammatical errors; (d) substituted words must result in a surprisal level greater than 20. The value of 20 was chosen arbitrarily as the standard since no prior studies have attempted to manipulate the word surprisal in the stimuli. Characteristics of the audiobook are shown in Table 2.

**Table 2.** Audiobook Characteristics.

Story	Word Count	Duration (min)	Word Rate (per min)	Average of Word Surprisal^a^	Word Surprisal of Altered/Original Words
run1_E45	2598	14.97	173.57	6.88	22.92
run1_control	2598	14.97	173.57	6.77	7.95
run2_E45	2381	14.79	160.94	7.03	22.83
run2_control	2381	14.79	160.94	6.92	9.11
run3_E45	2519	14.82	169.92	6.93	23.36
run3_control	2518	14.82	169.85	6.8	7.51
run4_E45	2577	14.7	175.26	6.66	22.27
run4_control	2577	14.7	175.26	6.55	7.3

a Average of word surprisal refers to the average surprisal value across all words within each story.

Audiobook text was aligned with the acoustic stimuli through forced alignment called Montreal Forced Aligner (McAuliffe et al., 2017) version 2.0.5. All stimuli were rerecorded at 44.1 kHz by a female native English speaker to ensure they sounded as natural as possible with the updated script. After each run, the experimenter asked five content questions about the story the participant had just heard to make sure they focused their attention on the story. All participants were able to answer at least four out of the five questions correctly. Audiobooks were presented at 70 dB SPL via a loudspeaker positioned directly in front of the participants at 0° azimuth and a distance of 0.8 m.

#### Signal Processing

##### Processing of EEG Data

Raw EEG data were processed in Brain Vision Analyzer (Brain Products, Gilching, Germany), where they were band-pass filtered between 0.1 and 40 Hz using a second-order Butterworth filter.

##### Extraction of Audio Features

Audio stimuli were recorded simultaneously with the EEG on an auxiliary channel to ensure optimal temporal alignment between the audio and EEG data. The audio soundtrack was then aligned offline with the recorded audio by calculating cross-covariance to identify the exact starting point of the soundtrack.

Speech envelopes were obtained by taking the absolute value of the Hilbert transform of the aligned audio soundtrack. These envelopes were then downsampled to match the EEG recording's sampling rate and attached to the EEG data for later analysis.

To obtain word onsets, we aligned the story transcripts to the acoustic stimuli using the Montreal Forced Aligner (McAuliffe et al., 2017) version 2.0.5, which was trained on the default English model LibriSpeech acoustic model and pronunciation dictionary (Panayotov et al., 2015). Word onset was represented as a binary time series that took the value of 1 at word onset and remained constant until the onset of the subsequent word (i.e., a step-like representation spanning the word duration).

**Figure 1.**
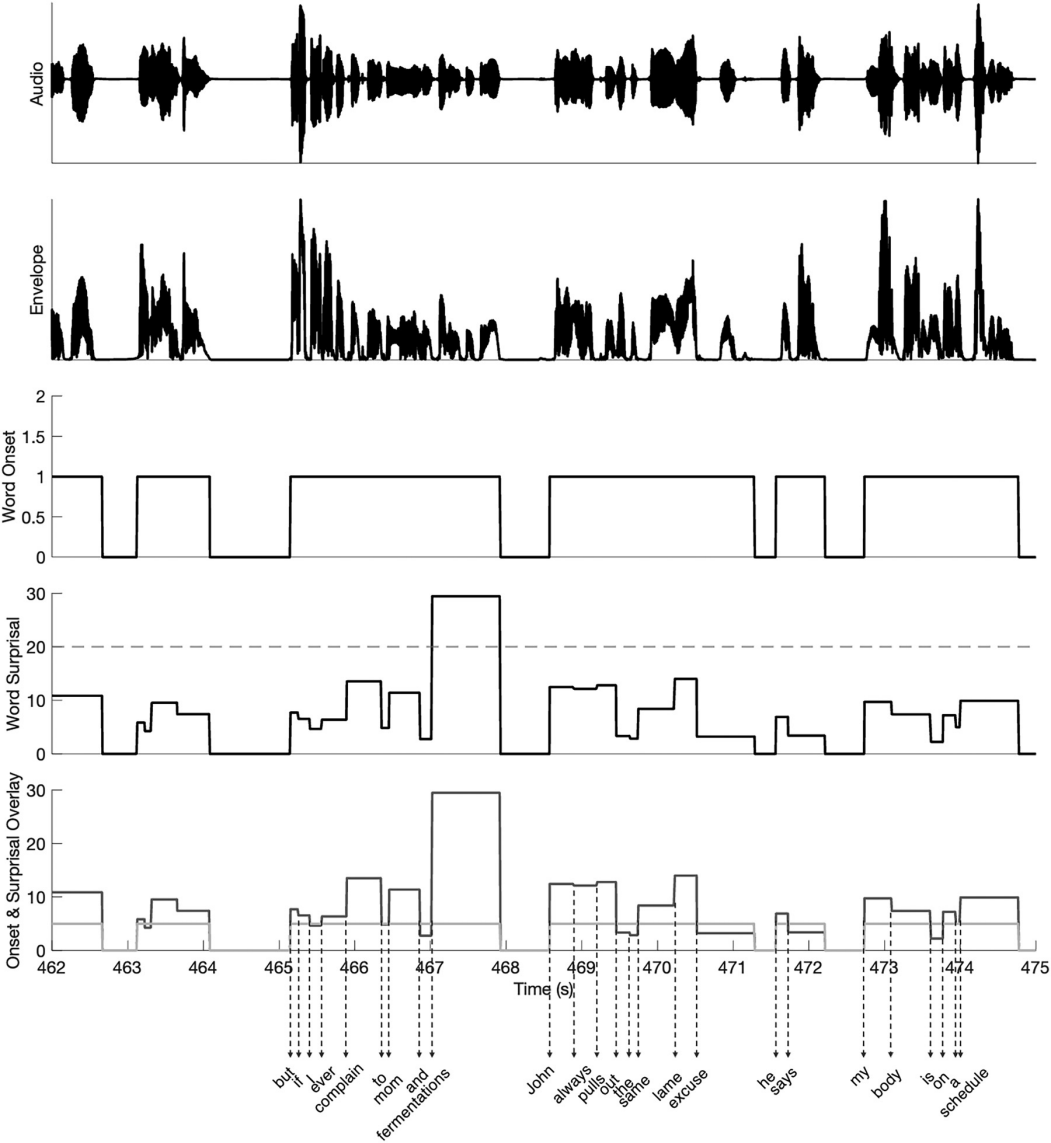
Segment of audio features.

Word surprisal was calculated as the negative logarithm of the conditional probability of a word given the four preceding words (equation (1)). Each word's surprisal value was assigned at its onset and held constant until the onset of the next word, resulting in a step function across the word duration rather than an impulse confined to a single time point. In the TRF design matrix, this step-like parameterization was adopted instead of an onset-locked impulse regressor because it yielded more stable and physiologically interpretable TRFs in our continuous-speech dataset. Mean surprisal value of E45 words is 22.94, and mean surprisal value of control words is 7.95. Figure 1 shows a 13-s segment of audio features from the E45 condition.

(1) $$S(w_{k})\;=-log_{2}(p(w_{k}|w_{k-4},\;w_{k-3},\;w_{k-2},\;w_{k-1}))$$

A 13-s segment of the audiobook in the E45 condition is shown, together with the acoustic and linguistic regressors used in the TRF analysis. From top to bottom: the raw audio waveform, the broadband temporal envelope, the word-onset regressor, and word surprisal values. Word onsets are modeled as onset-locked step functions spanning each word's duration. In the word-onset panel, some adjacent words appear as a single continuous step at 1 because the inter-word intervals are very short at this plotting scale; thus, a single visible step may contain multiple words rather than one prolonged word. Word surprisal (defined as the negative log probability of a word given the preceding four words) is likewise represented as a step function, with amplitude corresponding to the surprisal magnitude of each word. Surprisal was computed once at word onset and remains constant throughout the word; it is not updated continuously within a word. Although this step-like visualization places values across the full word duration, thereby increasing the apparent temporal granularity, it does not imply that surprisal evolves within the word itself. The dashed horizontal line in the surprisal panel indicates the threshold used to define high-surprisal words. The bottom panel overlays word onset (light gray; amplitude scaled up by five for visualization purposes only), word surprisal (dark gray), and the corresponding audiobook text to facilitate interpretation of feature alignment.

##### Artifact Removal

The EEG and audio were downsampled to 250 Hz after their alignment. Word onset and word surprisal were then aligned to avoid edge effects from downsampling. EEG and audio features were merged across conditions for each participant for later artifact removal. Any noisy channels with excessive noise and those near CI magnet and coil were excluded. Noisy channels were identified through visual inspection of the continuous data. Channels were classified as excessively noisy if they exhibited persistently high-amplitude fluctuations, poor signal quality throughout the recording, or prolonged flatlining relative to neighboring electrodes. On average, 2.05 channels per participant were excluded (*SD* = 1.2). Independent component analysis (ICA) was performed on the concatenated data to identify artifacts, including eye blinks, oculomotor movements, cardiac activity, and line noise. Components associated with these artifacts were manually removed after visually inspecting the component topographies. Previously removed channels were interpolated using spherical splines from neighboring channels (Perrin et al., 1989).

Additional preprocessing steps were taken to remove CI artifacts in CI users only. When auditory stimuli are present, the electrical stimulation and radio-frequency signals from cochlear implants introduce electrical stimulation artifacts into the EEG recordings (Wagner et al., 2018). Previous work in our lab has demonstrated the effectiveness of second-order blind identification (SOBI) in reducing CI-related artifacts (Belouchrani et al., 1997; Paul et al., 2020, 2021; Xiu et al., 2022), hence, we used the same approach to minimize CI artifacts in CI participants’ EEG datasets. In contrast to conventional ICA methods that primarily depend on higher-order statistical independence, SOBI separates sources by leveraging differences in their temporal autocorrelation structure. This feature is particularly beneficial for CI artifact removal, as stimulation artifacts display highly stereotyped temporal patterns that can be differentiated from neural activity based on their autocorrelation characteristics. SOBI has been validated in continuous speech paradigms and has demonstrated effective attenuation of CI-related artifacts while preserving underlying cortical responses. The cleaned EEG data were rereferenced to an average reference before further processing in preparation for TRF.

### Statistical Analysis

#### TRF

The processed audio and EEG signals were filtered using a 1–20 Hz bandpass, second-order, zero-phase filter. The filtered EEG and features were subsequently *z*-scored for each participant before TRF modeling using the mTRF toolbox 2.0 (Crosse et al., 2016) in MATLAB. Time lags between −100 and 1000 ms were selected for analysis. The mTRFcrossval function was employed to optimize the regularization parameter, minimizing overfitting of the speech envelope to the EEG data. This function uses leave-one-out N-fold cross-validation, estimating TRFs of all trials except one per participant. The estimated encoding model was then applied to predict the EEG signal for the left-out trial, and a Pearson's correlation coefficient was calculated to evaluate the match between predicted and actual EEG signals for each channel. This correlation was then averaged across channels to obtain a prediction accuracy for each TRF model.

The continuous EEG data were first epoched into 60-s trials prior to TRF calculation. We used a Tikhonov regularization, where the regularization parameter (*λ*) was optimized through an exhaustive search across a logarithmic range from 2^−5^ to 2^15^ within the training fold of each cross-validation iteration (Crosse et al., 2016). The correlation coefficients were then averaged across all participants, channels, and conditions for each regularization parameter, and the value associated with the highest correlation value was chosen as the optimal parameter to use. Epochs were then averaged for each condition, producing two TRF models (one per condition) for each participant. TRFs for each channel were baseline-corrected by subtracting the average value from the baseline period (−100 to −4 ms) from every point in the TRF waveform. The averaged and baseline-corrected TRF models were exported into a BESA avr format for later permutation testing.

To assess whether word surprisal added value, we tested whether prediction accuracy improved significantly when it was included in the model. Specifically, we compared the accuracy of a model using only envelope and onset (referred to as the acoustic model) with that of a model combining envelope, word onset, and word surprisal (referred to as the full model). A significantly higher prediction accuracy of the full model compared to the acoustic model would indicate that word surprisal is reliably tracked by the brain.

#### Cluster-Based Permutation Testing

We applied cluster-based permutation testing using BESA Statistics 2.0 software (Brain Electrical Source Analysis, GmbH, Germany) to compare the TRF models for surprisal of E45 and Control conditions in both CI and NH participants. The software automatically detects clusters in both time and space by running a series of corrected *t*-tests on TRF weights across all time points. Clusters are first identified when adjacent time points and neighboring electrodes (within a 4 cm channel diameter) show amplitude differences between conditions, with a cluster alpha level of 0.05. We used 1,000 permutations, and a Monte Carlo resampling procedure (Maris & Oostenveld, 2007) was applied to evaluate whether the observed clusters exceeded the predefined threshold. These results were then compared to a null distribution generated from random permutations of the data, thereby controlling for the multiple-comparisons problem.

#### TRF Peak Amplitude and Latency

To compare sensory processing between CI and NH, we focused only on the TRF model with the audio envelope as the regressor since it captured the acoustic feature of the stimuli. For sensory processing, we chose a region of interest (ROI) of six frontal-central channels (see Figure S1 in the Supplementary Material) and averaged the amplitudes across time per condition per participant. We identified the TRF-N1 and TRF-P2 components by extracting the latencies and amplitudes associated with the global minimum in the 50–150 ms time window and the global maximum in the 150–250 ms time window, respectively. The TRF-N1-P2 peak-to-peak amplitude was calculated by subtracting the TRF-N1 amplitude from the TRF-P2 amplitude.

For semantic processing analyses, we focused on the TRF model using word surprisal as the regressor. We selected an ROI of nine parietal channels (see Figure S1 in the Supplementary Material) and averaged the amplitudes across time for each condition and participant. TRF-N400 was isolated by subtracting the TRF model of the Control from that of the E45 condition. TRF-N400 peak latency was extracted based on the latency associated with the global minimum in the 200–1000 ms time window. TRF-N400 amplitude was computed as the mean amplitude across the ROI channels of the difference waveform within a 20 ms time window surrounding the peak latency in the 200–1000 ms interval.

#### WM and Semantic Judgment Accuracy

WM was measured as the number of words correctly recalled across all blocks, and semantic judgment accuracy was calculated as the proportion of correct sentence semantic judgments across all blocks. Independent-samples *t*-tests were used to compare word recall accuracy and semantic judgment performance between CI and NH participants.

#### Brain–Behavior Correlation

To understand whether people with greater WM have better speech comprehension, we correlated the recall accuracy in the listening span task and TRF-N400 amplitude. Spearman correlational analyses were performed using the *psych* package (Revelle, 2007) in R Studio between neural response data (TRF amplitude and latency), behavioral performance (WM, semantic judgment accuracy), and SSQ scores. The false discovery rate (FDR) method was applied to *p*-values to correct for multiple comparisons in the correlational analyses (Benjamini & Hochberg, 1995).

## Results

### Behavioral Results

Independent samples *t*-tests revealed that NH performed better in WM and semantic judgment in the listening span test, and reported higher ratings of SSQ than CI users (see Table 3). Two CI users and one NH participant had missing listening span data due to technical difficulties. Inspection of performance across blocks did not reveal a systematic decline over time, suggesting minimal fatigue effects (Figures S2 and S3 in Supplementary Material).

**Table 3.** Results of Listening Span Test and SSQ Ratings.

Behavioral Measures		CI	NH	*t*	*p*	Cohen's *d*
Listening span test						
	Word recalled (%)	40.9	62.8	−4.5	<.001	−1.44
	Sentence semantic judgment (%)	70.5	81.1	−2.9	.007	−0.92
SSQ						
	Speech (/10)	4.8	8.3	−6.9	<.001	−2.15
	Spatial (/10)	4	8.4	−8.2	<.001	−2.53
	Quality (/10)	6.3	9	−6.8	<.001	−2.10
	All (/10)	5	8.5	−8.6	<.001	−2.65

### TRF

#### Word Surprisal Was Reliably Tracked Within Story

**Figure 2.**
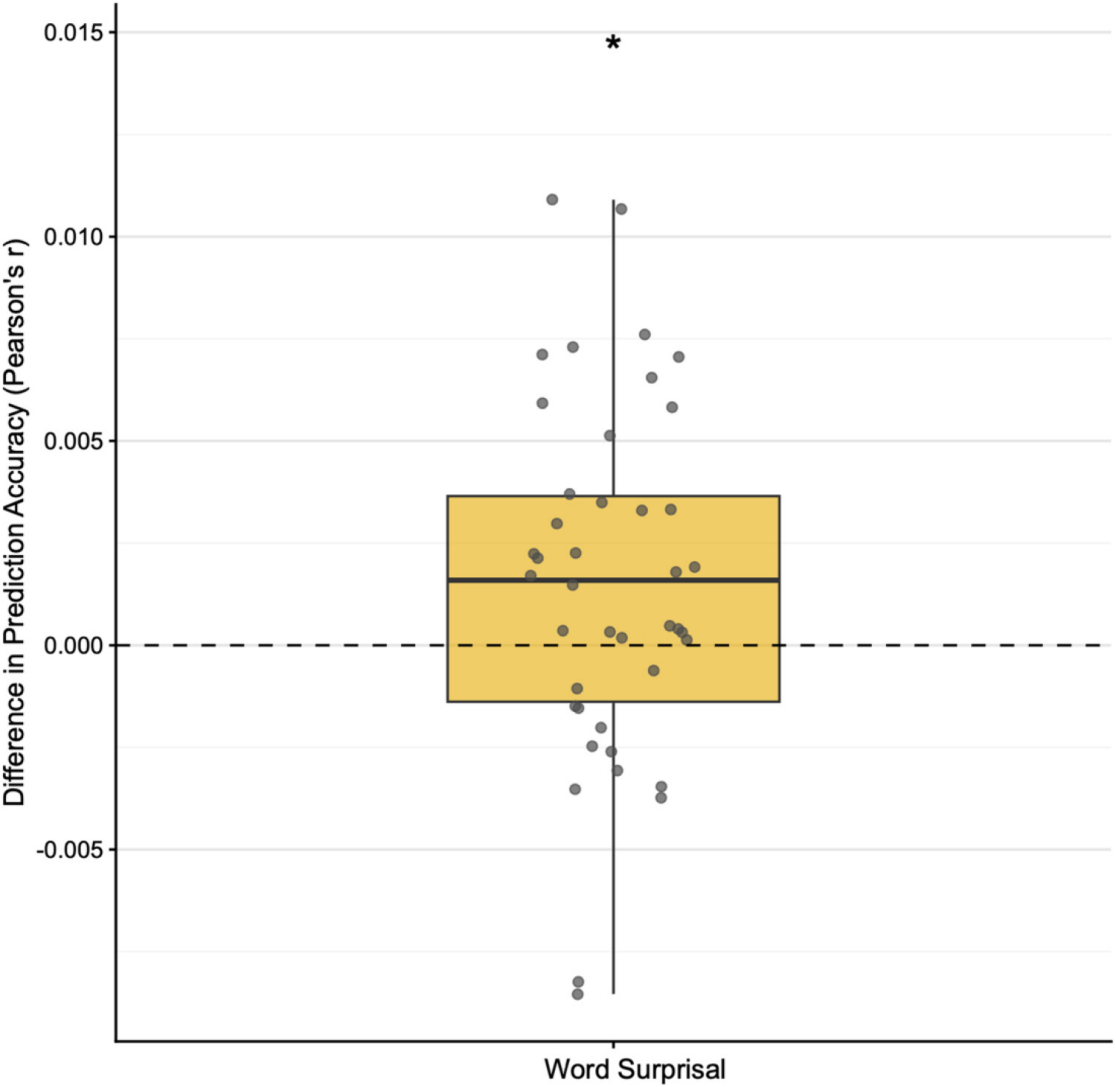
Added value of word surprisal. Increase in prediction accuracy attributable to word surprisal, computed as the difference between the full model and the acoustic model. The horizontal dashed gray line indicates the significance threshold for prediction accuracy. Individual delta prediction accuracy is shown in gray data points. *p < .05.

We conducted a repeated-measures ANOVA to assess the effects of hearing group (CI, NH), model (acoustic model, full model), and condition on prediction accuracy, demonstrating the added value of word surprisal (see Figure 2). There was a main effect of model, *F*(1, 40) = 5.16, *p* = .029, indicating a significantly higher prediction accuracy for the full model (mean accuracy in Pearson's *r* = 0.0422) compared to the acoustic model (mean accuracy in Pearson's *r* = 0.0407). Since no main effect of condition or hearing group was found, we averaged the prediction accuracy across conditions per participant. A one-sided Wilcoxon signed-rank test indicated that surprisal provided a significant added contribution beyond acoustic representations, as the median added value was greater than zero (*p* = .018). The effect size was small to moderate (Cohen's *d* = 0.35, 95% CI [0.04, 0.66]), suggesting a modest but reliable improvement in model performance when surprisal was included.

#### Sensory Processing (TRF of Envelope)

**Figure 3.**
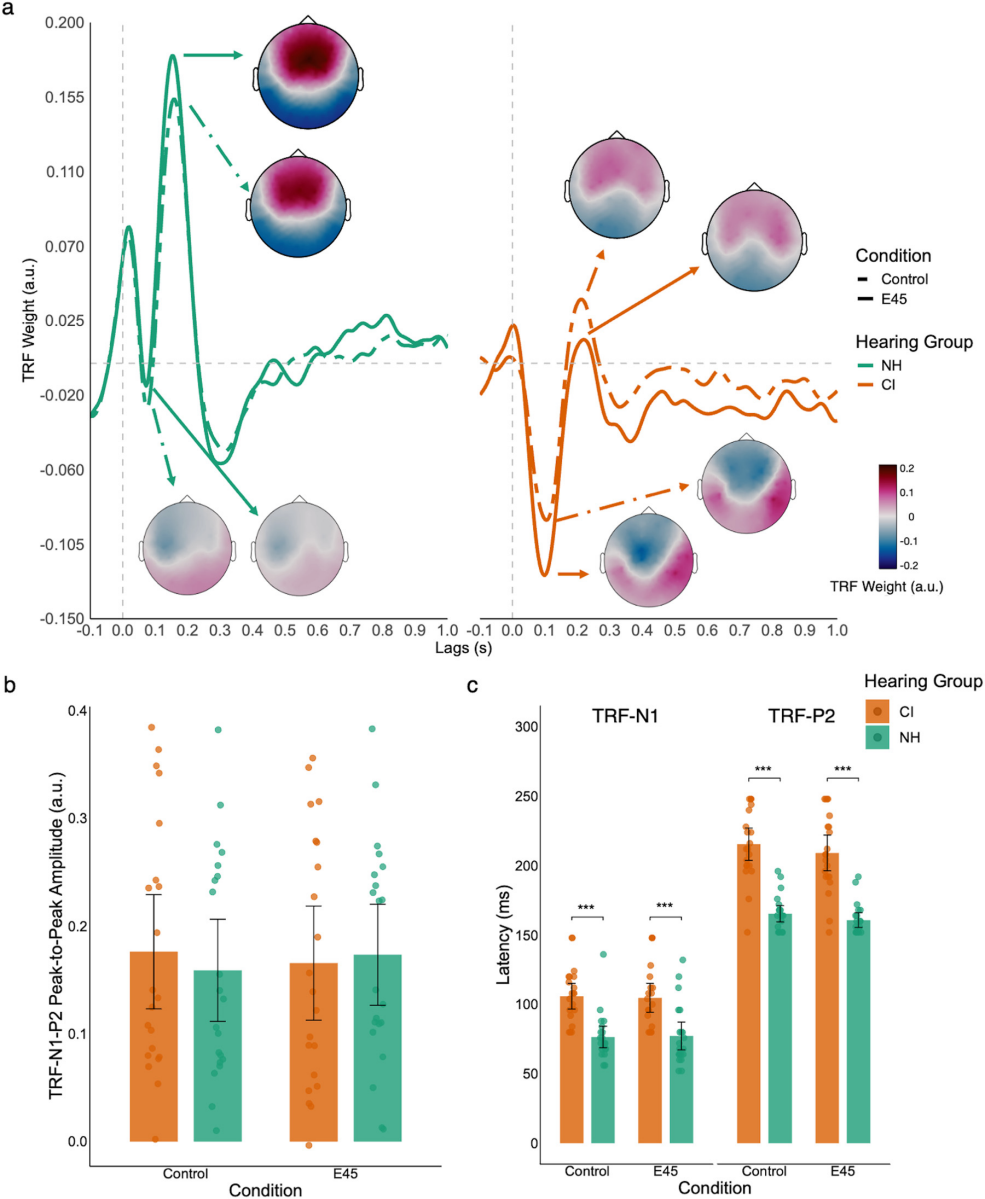
TRF-N1-P2 of speech envelope. Panel a: TRFs to the speech envelope for NH (green) and CI (orange) listeners in the Control (dashed lines) and E45 (solid lines) conditions. TRF waveforms to the speech envelope averaged across six central electrodes. Scalp topographies (topoplots) illustrate the spatial distribution of the N1 (negative peak) and P2 (positive peak) components at their respective latencies. Panel b: TRF-N1-P2 peak-to-peak amplitude across conditions and hearing groups. Bars represent group means, dots represent individual participants. Panel c: TRF-N1 and TRF-P2 latencies across conditions and hearing groups. Bars represent group means, dots represent individual participants. Error bars indicate 95% confidence interval. ***p < .001.

When averaging across six frontal-central electrodes, NH had a small but early TRF-N1 and a large TRF-P2 response, contrary to the big TRF-N1 and small TRF-P2 of CI (Figure 3(a)). Sensory responses between the Control and E45 conditions were generally similar for both hearing groups. We performed a cluster-based permutation test comparing TRF amplitude for the envelope between the E45 and Control conditions in each hearing group. The tests did not find any significant cluster.

*TRF Amplitude*. Given that there is no difference between the TRF-envelope amplitude of E45 and Control, we averaged the TRF weights across conditions for each participant. An independent *t*-test comparing the TRF-N1-P2 peak-to-peak amplitude revealed no significant difference between the two hearing groups, *t*(39.4) = 0.15, *p* = .885 (Figure 3(b)).

*Sensory TRF Latency*. We compared TRF-N1 and TRF-P2 latencies between hearing groups using two independent samples Welch *t*-tests. NH showed significantly earlier TRF-N1, *t*(39.9) = 5.28, *p* < .001, *d* = 1.63, 95% CI [0.92, 2.32], and earlier TRF-P2, *t*(28.2) = 9.19, *p* < .001, *d* = 2.84, 95% CI [1.96, 3.69] (Figure 3(c)).

#### Semantic Processing (TRF of Word Surprisal)

**Figure 4.**
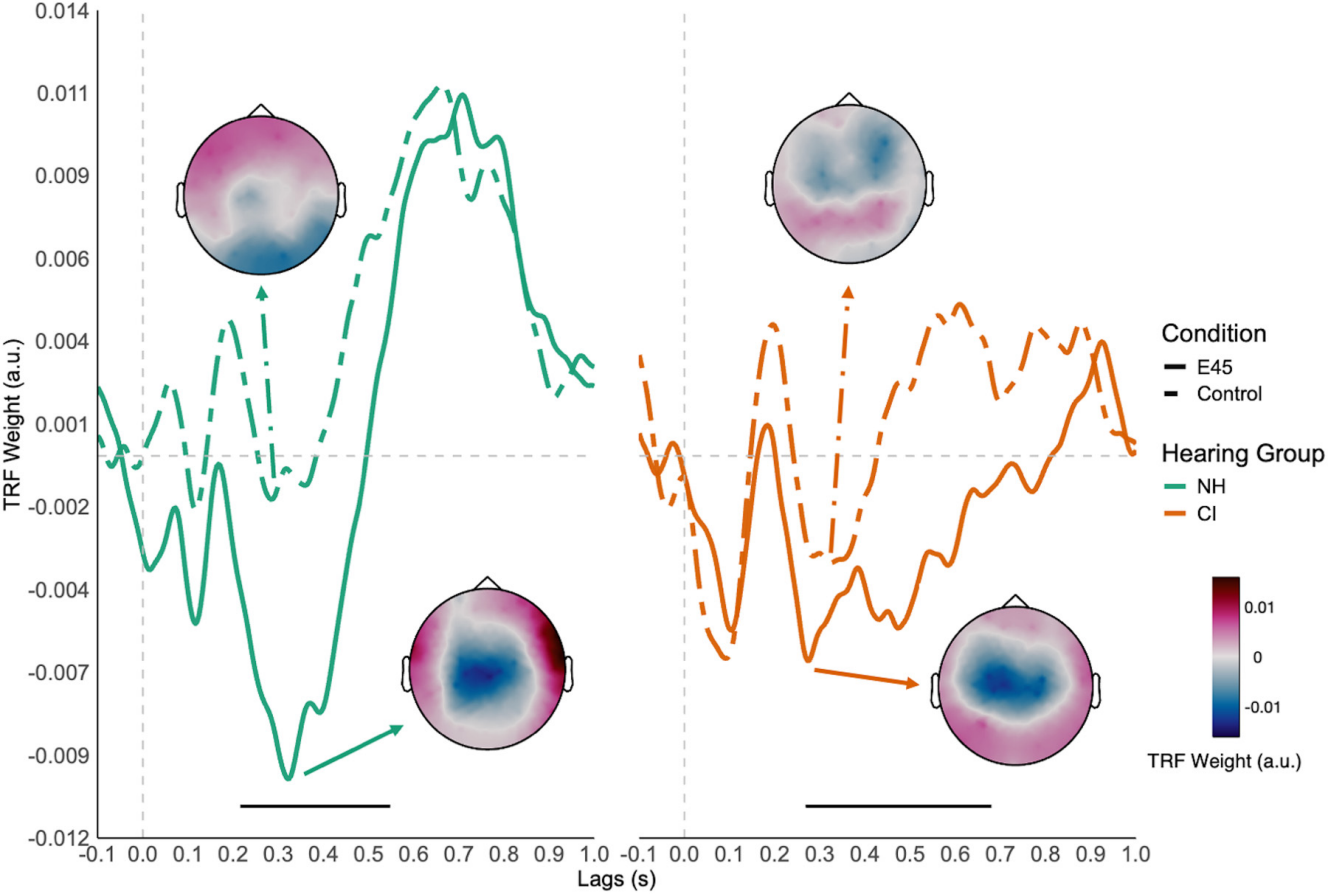
TRF-N400 of word surprisal. TRFs to word surprisal are shown for NH (green) and CI (orange) listeners in the Control (dashed lines) and E45 (solid lines) conditions. Waveforms represent averages across the electrodes within the significant cluster in each hearing group. Scalp topographies illustrate the spatial distribution of the TRF-N400 effect at the peak latency. Black horizontal bars indicate the significant time windows identified by cluster-based permutation testing.

*Within-Group TRF-N400 Effect*. Cluster-based permutation testing comparing TRF amplitude for word surprisal in the E45 and Control conditions revealed one significant cluster for both hearing groups. For CI users, a cluster of 16 channels across the frontal, central, and parietal regions showed greater negativity in the E45 than the Control condition from 268 ms to 680 ms, corrected *p*-value = .001. TRF amplitude of E45 was also significantly more negative at the central-parietal area than that of Control in NH from 216 ms to 548 ms, corrected *p*-value = .011 (Figure 4).

**Figure 5.**
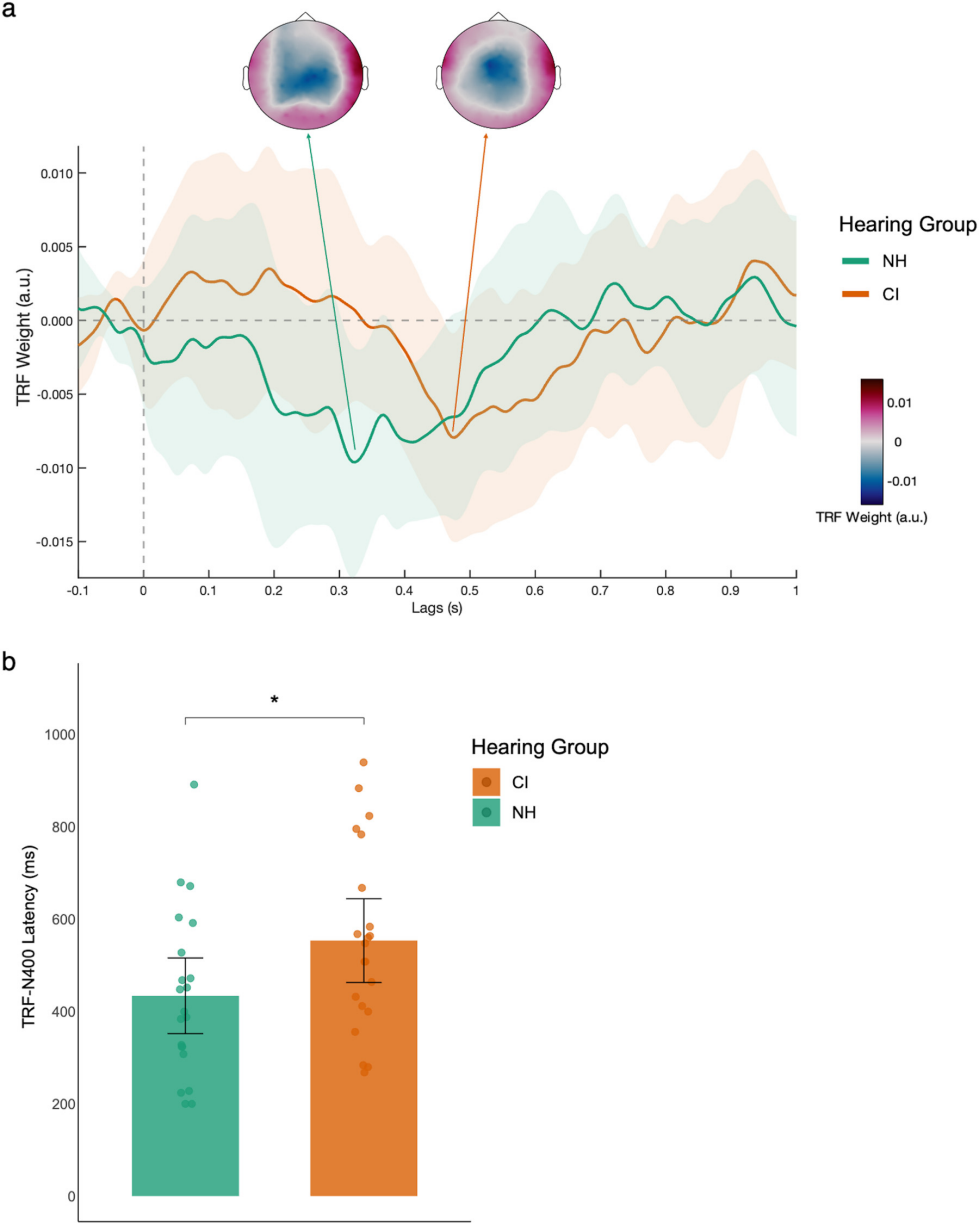
TRF-N400 difference wave between E45 and control conditions. Panel a: TRF-N400 difference wave (E45 minus Control) for NH (green) and CI (orange) listeners. Waveforms are averaged across nine parietal electrodes. Scalp topographies illustrate the spatial distribution of the difference effect at peak latency. Shaded areas represent 95% confidence intervals. Panel b: TRF-N400 peak latency for each hearing group. Bars represent group means, dots represent individual participants, and error bars indicate 95% confidence intervals. *p < .05.

*Between-Group TRF-N400 Effect*. A difference waveform was generated by subtracting the TRF model of Control from that of the E45 condition (Figure 5(a)). A permutation *t*-test did not find any significant clusters between the two hearing groups.

*TRF-N400 Latency.* Individual TRF-N400 peak latency was extracted from the difference waveform by taking the minimum TRF weight within a time window of 200 ms and 1000 ms. An independent samples *t*-test revealed that the TRF-N400 of NH (*M* = 427.2 ms) occurred significantly earlier than that of CI (*M* = 554.3 ms), *t*(37.75) = −2.29, *p* = .028, *d* = −0.71, 95% CI [−1.33, −0.08] (Figure 5(b)).

### Brain–Behavior Correlation

**Figure 6.**
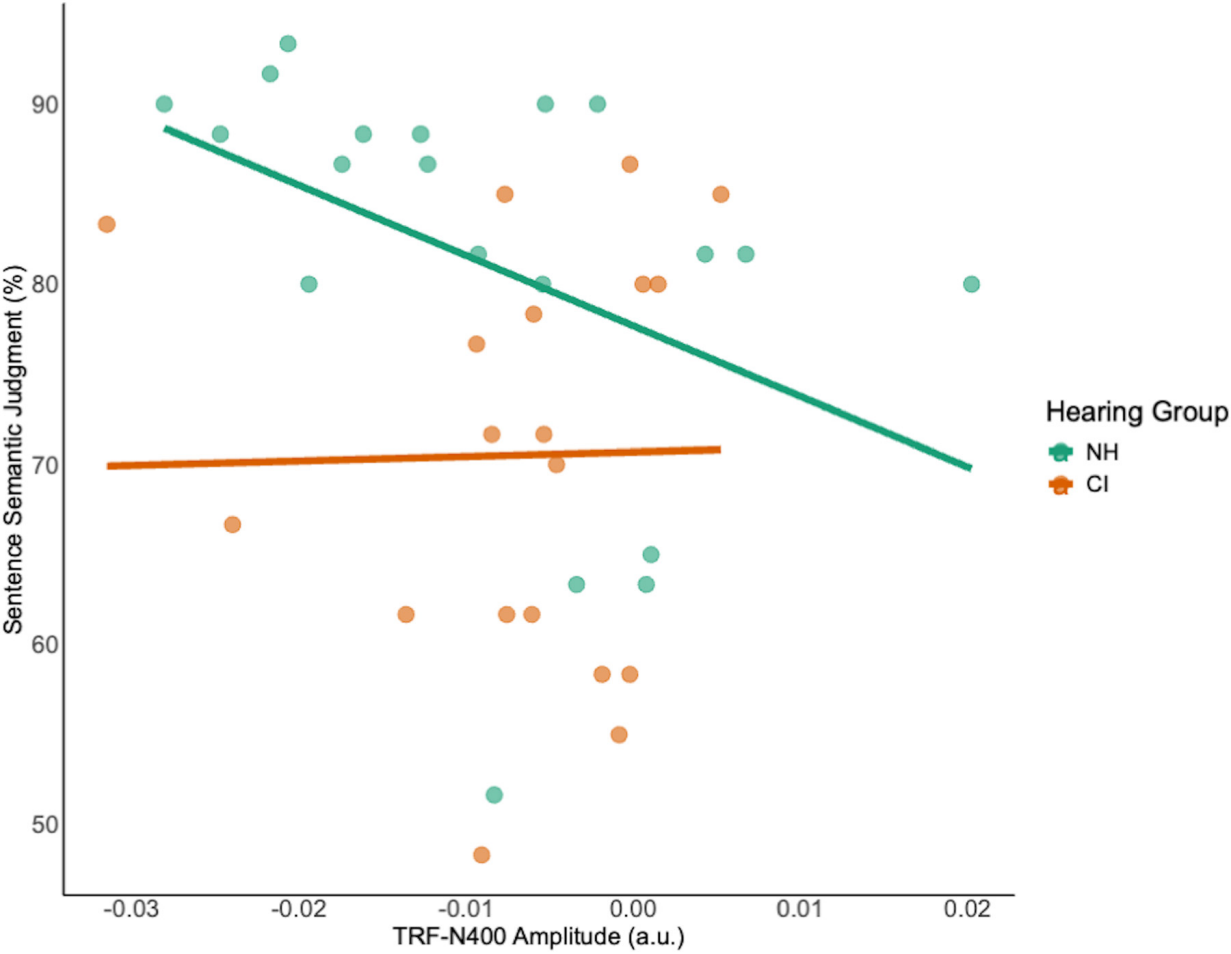
Correlation between behavioral semantic judgment accuracy and TRF-N400 amplitude. Scatterplots show the association between sentence semantic judgment accuracy (%) and TRF-N400 amplitude for NH (green) and CI (orange) listeners. Lines represent linear regression fits for each group. More negative TRF-N400 amplitudes indicate stronger neural responses.

Two CI users and one NH were unable to complete the behavioral listening span test, so their data were not included in the correlational analyses between behavioral measures (WM, semantic sentence judgment) and TRF-N400 response (amplitude and latency). For CI users, we did not find significant correlations between WM and TRF-N400 amplitude (ρ = −0.09, *p* = .715), WM and TRF-N400 latency (ρ = 0.24, *p* = .337), sentence semantic judgment and TRF-N400 amplitude (Figure 6, ρ = .2, *p* = .41), or sentence semantic judgment and TRF-N400 latency (ρ = −0.17, *p* = 0.5). For NH users, we did not find significant correlations between WM and TRF-N400 amplitude (ρ = −0.4, *p* = .081), WM and TRF-N400 latency (ρ = −0.03, *p* = .912), or sentence semantic judgment and TRF-N400 latency (ρ = 0.04, *p* = .859). However, a significant correlation was found between semantic judgment and TRF-N400 amplitude (Figure 6, ρ = –0.55, *p* = .011). We did not find any significant correlation between SSQ score and TRF-N400 amplitude (CI: ρ = 0.16, *p* = .514; NH: ρ = −0.2, *p* = .409) or TRF-N400 latency (CI: ρ = −0.05, *p* = .85; NH: ρ = 0.17, *p* = .495). Analyses examining associations between behavioral performance and age at implantation, duration of deafness, and chronological age did not reveal significant correlations (Figure S4 in the Supplementary Material).

## Discussion

This study aimed to determine whether semantic processing when listening to an audiobook is comparable between CI and NH, and if neural correlates of semantic processing are associated with behavioral measures. We validated results from previous ERP studies, which found a delayed N400 response in CI compared to NH (Deshpande et al., 2024; Finke et al., 2016; Hahne et al., 2012). In NH listeners, sentence semantic judgment performance was positively correlated with TRF-N400 amplitude, suggesting that the paradigm captures behaviorally relevant aspects of semantic processing. While associations with other behavioral measures were limited, the use of ecologically valid continuous speech provides an objective neural index of semantic processing that does not require explicit behavioral responses during EEG recording. However, successful implementation still depends on sustained attention, and further work is needed to establish the robustness and clinical applicability of this approach.

### Behavioral Results

In this study, NH participants showed higher performance in the listening span test, with significantly higher auditory WM and better sentence semantic judgment compared to CI users. This is consistent with previous studies that also compared auditory WM capacity between CI and NH (Prince et al., 2024; Tao et al., 2014), and people with or without hearing aid amplification (Doherty & Desjardins, 2015). It is important to note that the reduced audibility of CI users is unlikely to explain the difference, as all participants were allowed to adjust the volume to a comfortable level. They were also tested under their everyday listening conditions (i.e., best-aided hearing condition). The poorer behavioral semantic judgment of CI users is discussed below.

### Sensory Processing

We first compared the TRFs modeled with the audio envelope of E45 and Control within each hearing group to confirm whether there is a potential acoustic difference in the stimuli. The absence of a significant difference suggested that the observed effects between conditions were not attributable to low-level acoustic features of the conditions. Additionally, we also averaged the TRF models of the two conditions to only focus on the hearing group effect on sensory processing.

Consistent with previous studies, CI users showed a delayed sensory processing of TRF-N1 and TRF-P2 compared to NH (Burkhardt et al., 2022; Finke et al., 2015; Sandmann et al., 2015). In their longitudinal study, Sandmann et al. (2015) showed decreased N1 latencies in the bilateral auditory cortex during the first year after implantation, revealing cortical adaptations to the CI input. However, N1 latencies remained delayed compared to NH, even one year after implantation.

Although it is not the focus of the current study, we noticed the frontal-central TRF-N1 waveform (Figure 2, NH) did not resemble a typical ERP N1 response. Previous studies also show these enhanced early sensory responses. For instance, TRF-N1 was more positive when the SNR increased for both young and older NH participants (Herrmann, 2025a), with listening-in-quiet eliciting the most positive TRF-N1. In a MEG study (Chen et al., 2023), global field power of early MEG components that occurred at around 50–100 ms was greatly reduced when speech intelligibility was intact (Chen et al., 2023). Importantly, the elevated TRF-N1 in the current study is unlikely due to a lapse of attention or disengagement from the stimuli as all participants scored at least 80% correct in the content questions. Additionally, literature on TRF suggests that this enhancement of the TRF-N1 effect cannot be explained by attentional modulations (Herrmann, 2025b), but likely reflect reduced allocation of processing resources when auditory stimuli were more intelligible or familiar (Chen et al., 2023; Obleser & Kotz, 2011). When we performed a permutation test to compare sensory TRF amplitude differences between hearing groups, we observed significant clusters, which were the result of the latency shift of components between groups. Therefore, we chose to extract the TRF-N1-P2 amplitude, following the same approach as in Herrmann's study (2025a).

### Semantic Processing

#### Word Surprisal Was Tracked Over and Beyond Acoustic Features

Word surprisal had a significant added value compared with speech envelope and word onset, suggesting that this feature uniquely contributed to explaining neural responses. Previous studies using unaltered audiobooks showed the added value of word surprisal (Gillis et al., 2021; Weissbart et al., 2020). Our findings reveal similar results when using altered surprisal values in audiobooks. By parametrically altering surprisal values in the E45 condition, we elicited a strong and reliable TRF-N400 response that indexes processing of incongruent words and demonstrates the application of an objective measure of semantic processing in a clinical population.

#### Comparable Response Strength but Delayed Semantic Processing in CI Versus NH

Both groups showed a stronger TRF-N400 response in the E45 condition than in the Control condition, suggesting that altering the surprisal values can elicit the semantic incongruence effect.

We compared TRF-N400 amplitude and latency between CI and NH and found comparable amplitude, but a delayed latency in CI compared with NH. Previous studies using different paradigms to investigate the effect of hearing on semantic processing also show consistent patterns (Deshpande et al., 2024; Finke et al., 2016; Hahne et al., 2012). Since our CI participants were all experienced CI users (with more than one year of CI use), their auditory systems likely have adapted to the degraded CI input, which generally occurs during the first 3–6 months of CI use (Fu & Galvin, 2007). As discussed above, the CI provides limited spectral resolution compared to natural hearing, as the broad acoustic frequency range has to be compressed onto the limited length of the electrode array within the cochlea. This frequency compression and acoustic-to-electric mapping often lead to discrepancies between the peripheral neural activation patterns and the central speech patterns formed during normal hearing (Fu & Galvin, 2007). It is likely that such a degraded CI signal only partially maps to the lexical representations stored in long-term memory, therefore necessitating extra explicit processing of the limited information transmitted by the CI (Finke et al., 2015, 2016; Rönnberg et al., 2013; Stenfelt & Rönnberg, 2009). According to the ELU model (Stenfelt & Rönnberg, 2009), when the incoming speech signal does not match the mental lexicon, the brain must switch from fast, implicit processing to slower, explicit cognitive processing to resolve the ambiguity. Our observed N400 delay likely reflects this explicit reevaluation process. If CI listeners rely more heavily on relatively rigid lexical predictions, as reported in older adults by Broderick et al. (2021), then any discrepancy introduced by degraded auditory input would require additional time and cognitive resources to reconcile the incoming signal with stored lexical representations. In this framework, the prolonged TRF-N400 latency does not necessarily indicate weaker semantic activation, but rather a slower convergence process. Thus, when audibility is sufficient, CI users may ultimately achieve TRF-N400 amplitudes comparable to NH listeners, but require more time to reach that level of semantic integration.

#### Contextual Information Benefits CI Semantic Processing

Although we hypothesize that CI users would show a weaker semantic response than NH participants, we did not observe this result in our data. There are a few explanations. First, no background noise was added to the paradigm, which benefits the sensory and semantic processing. Background noise has been shown to further delay and reduce the N400 response (Burkhardt et al., 2022; Finke et al., 2016) as it likely burdens CI users when they must segregate target from distractors, and access lexical information with degraded CI input.

Additionally, the rich contextual information provided by the audiobook helped participants rule out word candidates more effectively compared to a single sentence without context. As participants were not informed that there would be unexpected words in the story, they might initially treat both conditions as the Control condition. Although each sentence in the audiobook varied in contextual constraints and cloze probability, the gist of the story and the context helped constrain the ending of a sentence, irrespective of conditions. It was proposed that the N400 reflects a stage of processing in which incoming stimuli temporarily synchronize with a broad, multimodal neural network shaped by context. This synchrony creates a dynamic, context-dependent conceptual representation that continues to be refined or remains stabilized as the meaning unfolds (Kutas & Federmeier, 2011), that is, the meaning of a stimulus emerges through time. At the time of the N400, the initial conceptual representations will be refined in the presence of a semantic anomaly. In the Control condition, participants constructed representations of congruous sentences without the need to constantly update, given the context constraints. As participants did not know the order of conditions, and even if they expected there would be incongruous words in the upcoming sentence, it was impossible to predict what the altered word would be. Hence, in the E45 condition, they may construct representations that code the congruency of sentences before they hear the altered word. Once they heard the surprising and incongruous word, its occurrence would then trigger the refinement of the conceptual representations. The rich contextual information provided uniquely by naturalistic speech, compared to other less ecologically valid paradigms, facilitates the formation of firm representations of congruous sentences for all participants, especially for CI users with degraded perceptual information. Previous studies have also supported the view that CI users utilize contextual information more frequently than NH individuals (Dingemanse & Goedegebure, 2019; O’Neill et al., 2019; Patro & Mendel, 2020). For instance, NH individuals benefited more from contextual cues in adverse listening conditions, whereas CI users benefited from semantic context only when the listening condition was less challenging (Patro & Mendel, 2020).

### Correlational Analyses

We observed a significant correlation between semantic judgment and TRF-N400 amplitude in NH, suggesting a reliable relationship between the neural semantic processing elicited by our paradigm and the behavioral measure. However, given the modest sample size, this pattern should be interpreted cautiously, and we do not claim a definitive dissociation between groups. Notably, when the two groups were pooled, the correlation was no longer significant, further underscoring the need for careful interpretation.

There are a few explanations for the lack of brain-behavior correlations in CI users. Although both EEG and the behavioral task involve semantic processing, the stimuli used in audiobooks provided richer contextual information than the behavioral task. Therefore, CI users were not able to use contextual cues to facilitate their judgment, thereby resulting in poorer overall behavioral performance. As a result, no consistent association was found. Given the lack of association between WM and TRF-N400, it is possible that listening to the audiobook without distractors was not very cognitively demanding for both hearing groups. Both CI and NH demonstrated good hearing-in-quiet performance; they were able to process the auditory stimuli relatively well. The ELU model suggests that under ideal listening conditions (Rönnberg et al., 2013), the mapping of phonological attributes to the lexical access is rapid and automatic. Although CI input is degraded, neural adaptation to spectral mismatch (Fu & Galvin, 2007) and potentially adaptive neural reorganization (Deshpande et al., 2024) help improve speech perception, which remains relatively fast and less demanding for additional explicit processing, especially when WM demand is low. However, the relationship between N400 and WM may be more obvious when a distractor or background noise is present. For instance, the study by Burkhardt et al. (2022) showed a positive correlation between N400 amplitude measured with background noise and WM in CI users, although the statistical result failed multiple comparison correction.

### Implications, Future Directions, and Limitations

This study substantiated an ecologically valid paradigm using naturalistic speech to assess semantic congruency, utilizing the TRF-N400 response. We showed that the TRF-N400 response strength is associated with behavioral semantic processing in NH. More ecologically valid stimuli simulate a naturalistic environment, helping to understand neural processing in everyday listening scenarios and striking a balance between experimental control and external generalizability. Additionally, using more naturalistic stimuli may engage participants and enable them to comply more effectively with the task requirements. Traditional ERP paradigms often repeat stimuli to improve signal quality, which can result in disengagement or lapses of attention that jeopardize the results and lead to fewer usable trials. This is particularly important for clinical populations (e.g., pediatric CI users or CI patients with comorbidities), who often have a reduced capacity to maintain attention over long durations. Hence, this ecologically valid paradigm can potentially be applied in clinical settings and provides an objective neural measure of semantic processing without requiring explicit behavioral responses.

A limitation of the current study is the imbalanced sample of hearing modality of CI users. A balanced and larger sample size would be helpful when attempting to study the effect of hearing modality (bilateral, unilateral, bimodal) on neural response, although we did not observe such an effect with the CI users in this study. It is also unclear whether manipulating the frequency of altered words would differentially affect the semantic processing of CI versus NH. As no prior studies adopted a similar approach, we chose a frequency of 45 s arbitrarily to strike a balance between the novelty of the altered word occurrence and a sufficient number of “trials” with such words. In a preliminary study involving 18 young NH adults, altered words presented every 175 s also elicited a clear TRF-N400 response; however, the amplitude did not significantly differ from that observed in the E45 condition. This finding suggests that the TRF-N400 response to contextual violations in continuous speech is relatively robust to variation in event density within this tested range. However, compared to the 175 s interval, the E45 condition yielded a greater number of critical target words, thereby increasing statistical power and stability of the estimated TRF-N400 response. For this reason, we adopted the E45 condition in the current study. Future work should systematically vary violation density to determine the optimal range that balances ecological validity, novelty, and neural sensitivity.

Another limitation is that the present study focused exclusively on lexical surprisal as an index of contextual predictability. While surprisal captures the improbability of an observed word given prior context, it does not reflect contextual uncertainty (entropy), which quantifies the dispersion of probability across possible upcoming words (Gillis et al., 2021). Because entropy and surprisal convey complementary information about predictive processing, modeling surprisal alone does not fully dissociate strong predictions from high-uncertainty contexts. Future studies should incorporate entropy measures to better characterize how contextual uncertainty and lexical prediction jointly shape neural responses during continuous speech comprehension.

Future studies should parametrically manipulate task difficulty in the audiobook listening paradigm by adding distractor noise at various levels, which further mimics the daily listening environment that people experience. Additionally, it is not clear whether other neurocognitive and linguistic factors are associated with the TRF-N400 response. In participants with Alzheimer's disease, N400 latency explains variance in a battery of neuropsychological tests (covering immediate memory, visuospatial skills, language, attention, and delayed memory) beyond clinical factors (Geiger et al., 2025). Factors such as inhibitory control (Moberly & Reed, 2019), nonauditory working memory (O’Neill et al., 2019), cognitive processing speed (Zucca et al., 2022), and demographic factors (Blamey et al., 2013; Holden et al., 2013; James et al., 2019; Skidmore et al., 2020) are associated with speech perception and comprehension. Although neurocognitive measures were collected in a few CI studies (Burkhardt et al., 2022; Kessler et al., 2020), they were not extensively used to correlate with neural response compared to speech perception measures. Future work could also examine the impact of using alternative language models to compute surprisal. Although the current study relied on a 5-gram model, recent transformer-based models can leverage much broader contextual information via attention mechanisms (Momenian et al., 2024; Xie et al., 2025). Systematically comparing surprisal estimates derived from traditional n-gram models and long-context neural language models would help determine whether incorporating discourse-level context improves the prediction of neural responses during continuous speech processing. Such comparisons could provide deeper insight into how different computational formulations of linguistic predictability map onto semantic processing in both CI and NH listeners.

## Conclusion

The present study used naturalistic audiobooks with altered word surprisal and observed semantic processing components shown in traditional ERP studies. The elicited effect is not attributed to the acoustic difference in the altered word. CI users showed delayed but comparable amplitude of semantic processing compared to NH. The amplitude of semantic processing at the neural level is associated with behavioral semantic judgment accuracy in NH, providing evidence that the paradigm can measure objective semantic processing. Ecologically valid stimuli thus offer an additional approach to clinical testing of speech comprehension in the clinical population. Future research should employ more comprehensive measures of neurocognitive and linguistic measures to uncover factors associated with neural semantic processing in CI users.

## Supplemental Material

sj-docx-1-tia-10.1177_23312165261439202 - Supplemental material for Electrophysiological Assessment of Semantic Processing of Cochlear Implant Users Using an Audiobook Supplemental material, sj-docx-1-tia-10.1177_23312165261439202 for Electrophysiological Assessment of Semantic Processing of Cochlear Implant Users Using an Audiobook by Shimin Mo, Claude Alain, Kristen E. Li and Andrew Dimitrijevic in Trends in Hearing

sj-docx-2-tia-10.1177_23312165261439202 - Supplemental material for Electrophysiological Assessment of Semantic Processing of Cochlear Implant Users Using an Audiobook Supplemental material, sj-docx-2-tia-10.1177_23312165261439202 for Electrophysiological Assessment of Semantic Processing of Cochlear Implant Users Using an Audiobook by Shimin Mo, Claude Alain, Kristen E. Li and Andrew Dimitrijevic in Trends in Hearing
