# Supplementary material for: Electrophysiological Assessment of Semantic Processing of Cochlear Implant Users Using an Audiobook
Source: Trends Hear. 2026 Apr 27;30:23312165261439202. doi: 10.1177/23312165261439202 (PMC13133447; doi:10.1177/23312165261439202)
Supplement: sj-docx-1-tia-10.1177_23312165261439202 - Supplemental material for Electrophysiological Assessment of Semantic Processing of Cochlear Implant Users Using an Audiobook [file sj-docx-1-tia-10.1177_23312165261439202.docx]

Supplementary Materials

Figure S1. Parietal (left) and Central (right) ROIs


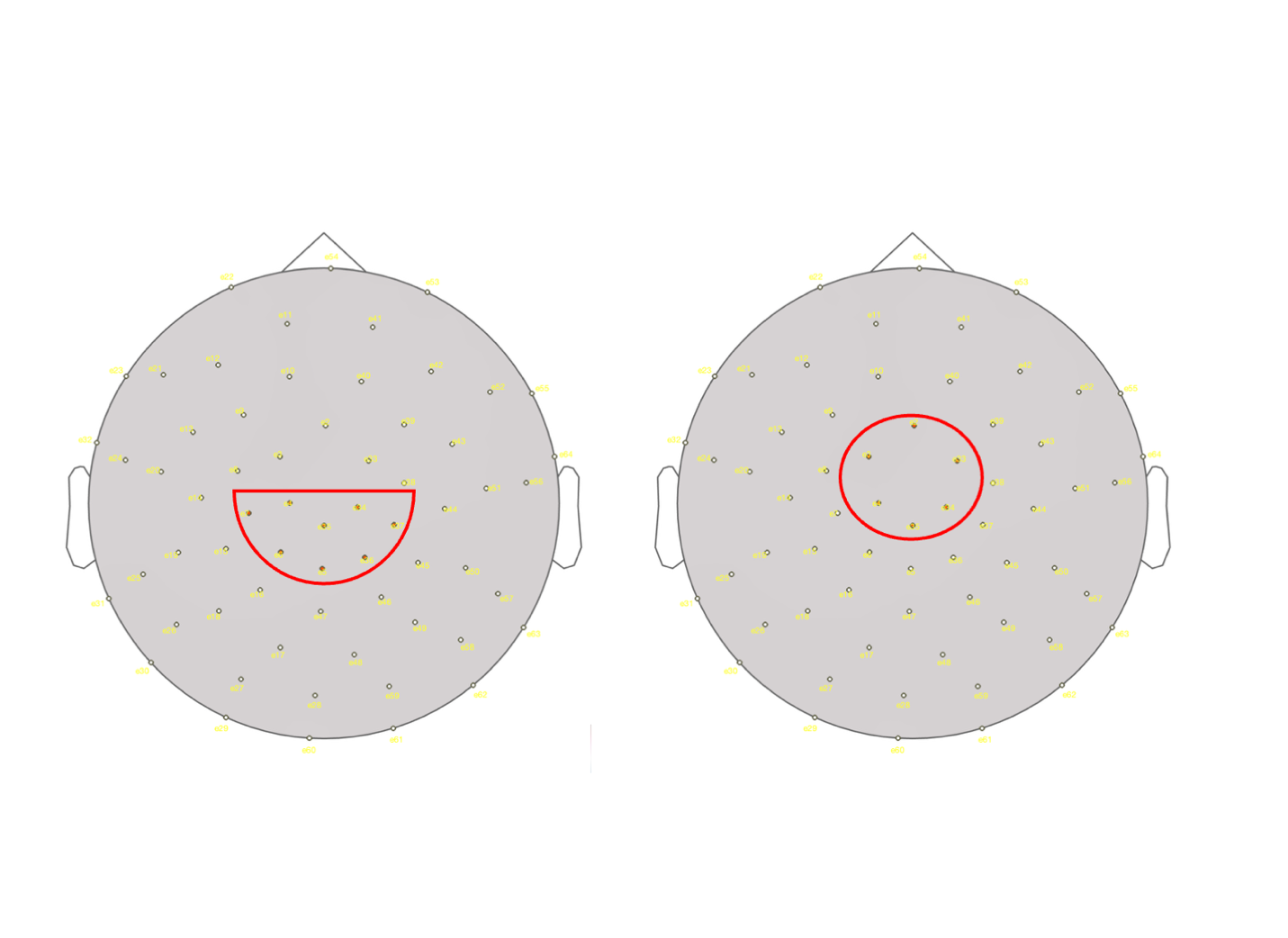


Figure S2. Behavioural Sentence Semantic Judgment Across Blocks


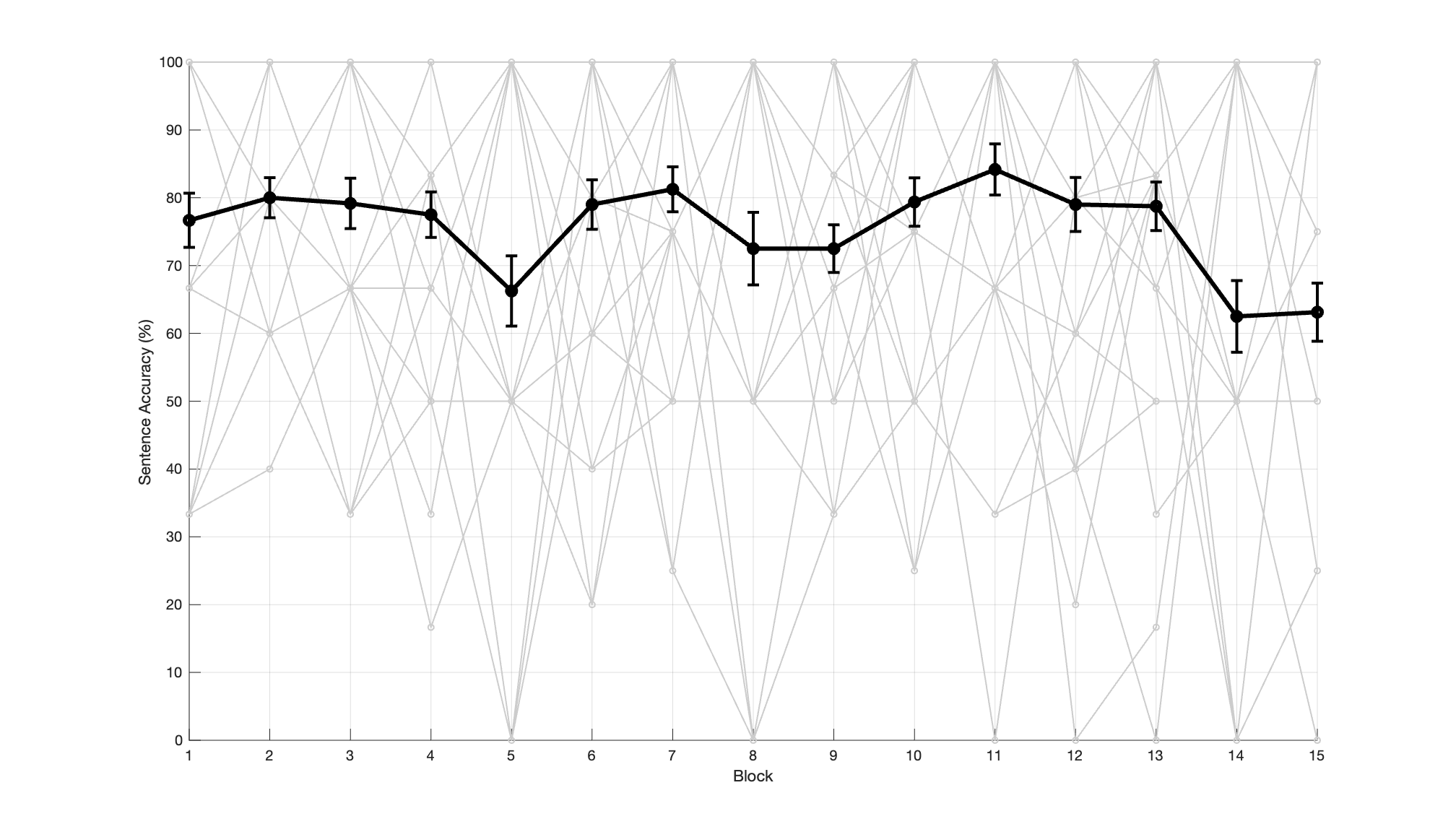


*Note*. Individual data is shown in gray. Error bar = SEM. A linear mixed-effects model including both linear and quadratic block terms revealed significant effects of block order (linear: β = −0.54, SE = 0.23, *t* = −2.35, *p* = .019; quadratic: β = −0.13, SE = 0.06, *t* = −2.26, *p* = .025). Model comparison confirmed that inclusion of the quadratic term significantly improved model fit (χ²(1) = 5.08, *p* = .024), indicating a non-monotonic pattern across blocks rather than a steady decline consistent with fatigue.

Figure S3. Behavioral Word Recall Accuracy Across Blocks


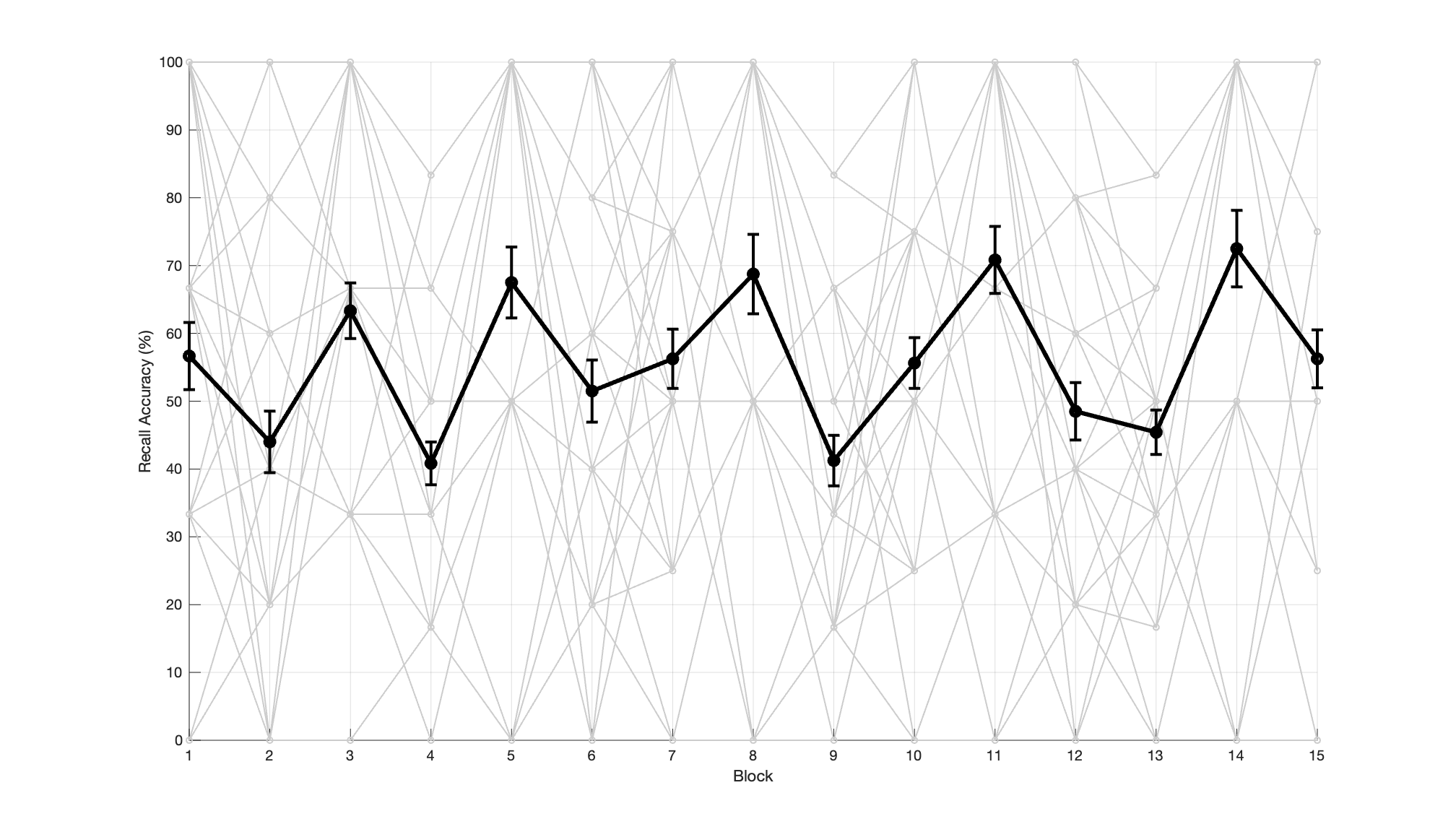


*Note*. Individual data is shown in gray. Error bar = SEM. A linear mixed-effects model showed that block order did not significantly predict recall performance (*β* = 0.40, SE = 0.23, t = 1.71, *p* = .087), indicating no evidence of progressive deterioration across the session.

Figure S4. Correlation Matrix of TRF-N400 and Demographic Factors


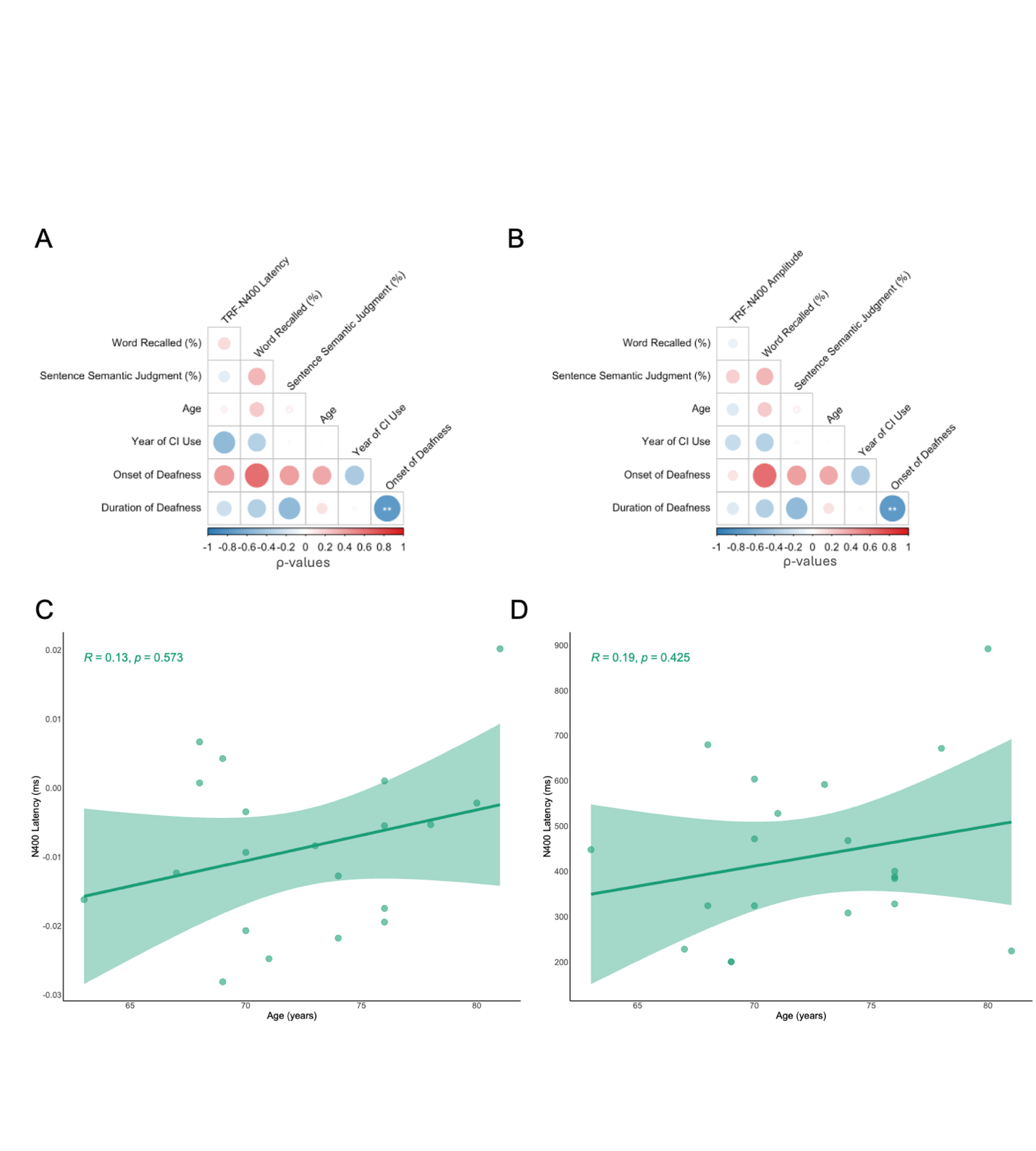


*Note*. Panel A: correlation matrix of TRF-N400 latency of CI users with behavioral measures and demographic factors. Panel B: correlation matrix of TRF-N400 amplitude of CI users with behavioral measures and demographic factors. Panel C: correlation of NH’s TRF-N400 latency and age. Panel D: correlation of NH’s TRF-N400 amplitude and age. ***p* < .01; shaded area = SE.
